# Atherosclerosis Residual Lipid Risk-Overview of Existing and Future Pharmacotherapies

**DOI:** 10.3390/jcdd11040126

**Published:** 2024-04-21

**Authors:** Muntaser Omari, Mohammad Alkhalil

**Affiliations:** 1 Cardiothoracic Centre, Freeman Hospital, Newcastle-upon-Tyne NE7 7DN, UK; muntaser.omari@nhs.net; 2 Translational and Clinical Research Institute, Newcastle University, Newcastle-upon-Tyne NE1 7RU, UK

**Keywords:** atherosclerosis, lipid, LDL-c, HDL-c, triglycerides, Lp(a), residual risk

## Abstract

Patients with atherosclerotic disease remain at increased risk of future events despite receiving optimal medical treatment. This residual risk is widely heterogeneous, but lipoprotein particles and their content play a major role in determining future cardiovascular events. Beyond low-density lipoprotein cholesterol (LDL-c), other lipoprotein particles have not demonstrated similar contribution to the progression of atherosclerosis. Statins, ezetimibe, and more recently, proprotein convertase subtilisin kexin 9 (PCSK9) inhibitors and bempedoic acid have confirmed the causal role of LDL-c in the development of atherosclerosis. Data on high-density lipoprotein cholesterol (HDL-c) suggested a possible causal role for atherosclerosis; nonetheless, HDL-c-raising treatments, including cholesteryl-ester transfer protein (CETP) inhibitors and niacin, failed to confirm this relationship. On the other hand, mendelian randomisation revealed that triglycerides are more implicated in the development of atherosclerosis. Although the use of highly purified eicosapentaenoic acid (EPA) was associated with a reduction in the risk of adverse cardiovascular events, this beneficial effect did not correlate with the reduction in triglycerides level and has not been consistent across large phase 3 trials. Moreover, other triglyceride-lowering treatments, such as fibrates, were not associated with a reduction in future cardiovascular risk. Studies assessing agents targeting angiopoietin-like 3 (lipoprotein lipase inhibitor) and apolipoprotein C3 antisense will add further insights into the role of triglycerides in atherosclerosis. Emerging lipid markers such as lipoprotein (a) and cholesterol efflux capacity may have a direct role in the progression of atherosclerosis. Targeting these biomarkers may provide incremental benefits in reducing cardiovascular risk when added to optimal medical treatment. This Review aims to assess available therapies for current lipid biomarkers and provide mechanistic insight into their potential role in reducing future cardiovascular risk.

## 1. Introduction

Despite advances in revascularisation strategies as well as pharmacotherapies, acute coronary syndrome is one of the leading causes of morbidity and mortality in the world [1]. One in five patients who survive acute coronary syndrome presentation returns with a second event within the following five years despite being on the guidelines-directed optimal medical therapy [2,3]. This residual risk is rather heterogeneous and not identical among patients re-presenting with a secondary ACS. Therefore, characterising the risk at an individual level may allow tailored therapy and precise intervention to reduce future cardiovascular events. The use of blood, imaging, or genomic biomarkers may enable us to understand future risks and offer patients intensive therapies accordingly [4,5,6,7]. This is important given the recent advances in pharmacotherapy, particularly in the field of lipidology.

The intricate relationship between lipoprotein particles and their content plays a significant role in determining future cardiovascular events. The role of low-density lipoprotein cholesterol (LDL-c) was recognised early, and recent studies have confirmed its causal relationship with the development of atherosclerotic disease [8,9,10,11]. However, data on other lipoprotein particles, such as high-density lipoprotein (HDL) or triglycerides, were less consistent, and their direct role in reducing future cardiovascular risk could not be ascertained [12,13,14,15]. Nonetheless, new triglyceride-lowering treatments targeting angiopoietin-like 3 (lipoprotein lipase inhibitor) and apolipoprotein C3 antisense will add further insights into the role of triglycerides in the progression of atherosclerosis [16].

Lipoprotein (a) and cholesterol efflux capacity are emerging as a therapeutic target for patients at high risk of future cardiovascular events. Targeting these biomarkers may provide incremental benefits in reducing cardiovascular risk when added to optimal medical treatment. This Review aims to assess available therapies for current lipid biomarkers and provide mechanistic insight into their potential role in reducing future cardiovascular risk.

## 2. Low-Density Lipoprotein Cholesterol Lowering Treatment

Numerous primary and secondary prevention trials have consistently shown a strong and direct correlation between LDL-c levels and atherosclerotic cardiovascular disease risk [17]. LDL and other apoB-containing particles are required to initiate and propagate atherosclerotic plaques [18]. Retention and accumulation of apoB lipoproteins (>90% are LDL particles) in the arterial wall initiate atherosclerotic plaque formation [19]. Correspondingly, the benefit of lipid-lowering therapies is proportional to the degree of reduction in apoB, and this benefit appears to increase over time [20]. Therefore, guidelines have recommended targets for reducing LDL-c with a lower threshold for patients who are considered at very high risk or recurrent events [21].

Statins are the cornerstone of preventing and treating atherosclerotic disease. Statins inhibit the rate-limiting step of cholesterol biosynthesis, hydroxymethylglutaryl (HMG)-CoA reductase and block the endogenic cholesterol synthase pathway, up-regulating hepatic LDL receptors (LDLR) expression, resulting in lower plasma LDL-c. The reduction in cardiovascular risk is proportionate to the decrease in LDL-c. For every 1 mmol/L reduction in LDL-c, there is a 22% relative risk reduction in future cardiovascular events [17]. Moreover, the benefits were more apparent in patients with high baseline clinical risk, such as post-coronary artery bypass grafts or polyvascular disease [22,23]. Statins are known to have pleiotropic features, including anti-inflammatory and plaque stabilisation effects [24,25]. There are some variations in the pharmacodynamic properties among different statins (Table 1). Mechanistically, it is presumed that lipid evacuation and replacement with fibrosis, in response to lowering LDL-c, is the plausible statin mechanism [4]. This would render the plaque more stable and less likely to rupture and cause future events. Statins are usually well-tolerated. Myalgia remains the most common side effect of statin use, with documented rates from 1 to 10%. Rhabdomyolysis, on the other hand, is the most serious adverse effect, but its occurrence is rare (less than 0.1%). There are a few conditions that may increase the risk of rhabdomyolysis, which include hypothyroidism, alcohol abuse and poly-pharmacology. Liver function derangement is not uncommon and occurs in about 1%. There is an increased risk of diabetes associated with statins, but this risk is small, and the benefits of statins significantly outweigh this risk [26]. Additionally, statins are associated with a paradoxical increase of reductase protein that may attenuate their efficacy and reduce their safety. Degradation of reductase protein (HMG-CoA reductase degrader) would eliminate this risk. The recent development of compounds that share structure-activity with sterol analogues may help with this phenomenon, lower cholesterol, and reduce atherosclerotic plaques in experimental studies [27].

The main driver of clinical benefits in response to statin treatment has always been debated [28]. The statin versus LDL-c hypothesis seems to have shifted with the results of ezetimibe in the Improved Reduction of Outcome: Vytorin Efficacy International Trial (IMPROVE-IT) [11]. Ezetimibe reduces intestinal cholesterol absorption by targeting the Niemann-Pick C1-like 1 (NPC1L1) protein. It is worth noting that the absorption of ezetimibe differs according to age, and it is higher in older adults [29]. It is associated with an 18–22% reduction in LDL-c, which resulted in a 2% absolute risk reduction in patients presenting with acute coronary syndrome when combined with simvastatin after a median follow-up of 6 years [11]. The Plaque Regression With Cholesterol Absorption Inhibitor or Synthesis Inhibitor Evaluated by Intravascular Ultrasound (PRECISE-IVUS) study showed greater plaque regression with the combination of ezetimibe and atorvastatin compared to atorvastatin only [30]. Ezetimibe is well-tolerated and has a good safety profile, with few to no adverse effects reported in the literature.

More recently, inhibiting proprotein convertase subtilisin–kexin type 9 with monoclonal antibodies using evolocumab and alirocumab has been added to the LDL-c reduction armamentarium. PCSK9 is responsible for extracellular binding and subsequent degradation of LDLRs, which stops LDL clearance from circulation. Monoclonal antibodies targeting PCSK9 maintain the number of LDLRs and subsequently lead to a reduction in LDL-c. Large phase 3 outcome studies showed a significant decrease in LDL-c, which was translated into a significant reduction in adverse clinical outcomes [31,32]. The Further Cardiovascular Outcomes Research with PCSK9 Inhibition in Subjects with Elevated Risk (FOURIER) reported a 59% reduction in LDL-c using evolocumab on top of statin treatment. This led to a 1.5% absolute risk reduction in major adverse cardiovascular events over 2.2 years of follow-up in patients with stable atherosclerotic disease [31]. Similarly, the use of alirocumab in the ODYSSEY OUTCOMES trial resulted in a 1.6% absolute risk reduction after 2.8 years of follow-up [32]. Interestingly, there was a mortality benefit associated with the use of alirocumab, which may reflect the high-risk nature of the targeted cohort of acute coronary syndrome as opposed to stable patients in the FOURIER study. Both monoclonal antibodies are safe, with the most common adverse effect being a mild injection site reaction (itching, rash and swelling). There is no evidence to suggest worsening cognitive function with the use of PCSK9i despite achieving very low LDL-c [33]. One limitation to the routine use of PCSK9i is related to its costs, and various strategies were implemented to improve patient’s access to this promising treatment [34].

Inclisiran is a long-acting synthetic small interfering RNA inhibitor that selectively and catalytically silences the translation of PCSK9 mRNA in the hepatocytes. It is administered subcutaneously once every 6 months, but initially, early administration of three months following the first injection is required to boost the LDL-c reduction effect. This provides an advantage over monoclonal antibodies when inhibiting PCSK9 and may help overcome issues related to adherence to lipid-lowering therapies [35]. Several trials have effectively demonstrated that adding inclisiran to the maximum tolerated statin has resulted in a significant reduction in LDL-c, up to 50% [36]. Notably, the magnitude of LDL-c reduction with inclisiran is slightly lower than that of inhibiting PCSK9 using monoclonal antibodies [36]. Whether reducing LDL-c using inclisiran will translate into better clinical outcomes is yet to be determined. Like evolocumab and alirocumab, inclisiran is associated with infrequent side effects and predominately are related to local reactions at the administration site alongside muscular pain. Other side effects, such as upper respiratory tract symptoms, have also been reported with its use [37].

Bempedoic acid is the latest LDL-c-specific lowering treatment. It is an oral prodrug, and its metabolites target ATP-citrate lyase (ACL), an enzyme that is upstream from HMG coenzyme A reductase, the enzyme inhibited by statins [38]. Bempedoic acid provides a potential alternative for patients who are statin intolerant, but it could also be used in combination with statins [38,39]. Several studies highlighted its effect on lowering LDL-c either in combination with maximally tolerated statins or when used alone in statin-intolerable patients [40,41,42,43,44]. The CLEAR (Cholesterol Lowering via Bempedoic Acid [ECT1002], an ACL-Inhibiting Regimen) Outcomes trial demonstrated that bempedoic acid was associated with a 21% decrease in LDL-c after 6 months of treatment compared with placebo in patients who were intolerant to statins who had or were at high risk for, cardiovascular disease. Such difference in LDL-c has led to a 13% relative risk reduction in the incidence of cardiovascular death, nonfatal myocardial infarction, nonfatal stroke, or coronary revascularisation when compared to placebo [44]. Importantly, there was an increase in the incidence of gout, cholelithiasis, and small derangements in serum creatinine and liver enzymes associated with the use of bempedoic acid compared to the placebo group [44]. The effect of bempedoic acid on creatinine has been small, stable and consistent regardless of baseline renal function. This is presumed to be related to the inhibition of tubular reabsorption of creatinine, although bempedoic acid has also been associated with small changes in urea, which appears early and is stable over time irrespective of baseline renal function [45]. Using another biomarker, such as cystatin C which is a muscle mass-independent marker, may provide more insights into the role of bempedoic acid on renal function [46].

Overall, the reduction in LDL-c, irrespective of the mechanism of the used drug, was consistently associated with improved clinical outcomes. Nonetheless, a significant proportion of patients remained at increased risk despite achieving an ‘optimal’ LDL-c level. Therefore, there was a rationale to target other lipoprotein particles aiming to reduce lipid-related atherosclerotic risk.

## 3. High-Density Lipoprotein Cholesterol Raising Treatment

Low plasma level of high-density lipoprotein cholesterol (HDL-c) is a recognised risk marker in patients with previous and recurrent cardiovascular event [47]. Data from the Framingham Heart Study highlighted a unique association between low HDL-c and cardiovascular death with less correlation with other causes of deaths [48]. However, subsequent Mendelian randomisation challenged the concept that raising HDL-c would translate into lowering cardiovascular risk [49]. This finding matched the results of pharmacotherapies aiming to increase HDL-c.

Cholesteryl ester transfer protein (CETP) inhibitors were used early to reduce future cardiovascular risk, with the aim of increasing HDL-c levels. CETP facilitates the exchange of triglycerides and cholesteryl ester between high-density lipoprotein (HDL) and apoB100-containing lipoproteins [50]. Evidence from genetic studies highlighted that variants in the *CETP* gene were associated with higher levels of HDL-c, lower LDL-c, and lower risk of coronary artery disease [50]. Therefore, inhibiting CETP may provide a similar lipid profile, which may translate into better clinical outcomes. The first CETP inhibitor, torcetrapib, was associated with a 25% increased risk of cardiovascular events despite achieving high HLD-c and low LDL-c levels [13]. The failure of this agent was thought to be related to off-target effects, including a rise in blood pressure and aldosterone levels [13]. Importantly, torcetrapib did not reduce the progression of coronary atheroma in the Investigation of Lipid Level Management Using Coronary Ultrasound to Assess Reduction of Atherosclerosis by CETP Inhibition and HDL Elevation (ILLUSTRATE) trial [51]. Nonetheless, all subsequent CETP inhibitors failed to show any incremental benefits in reducing clinical outcomes [3,4,52]. Anacetrapib was associated with a modest 9% relative risk reduction in major coronary events in patients with atherosclerotic vascular disease who were receiving intensive statin therapy. However, this effect was likely driven by the reduction in LDL-c of 18% associated with its use [12]. Obicetrapib is the newest CETP inhibitor and appears to be the most potent one by increasing HDL-c by 165% and reducing LDL-c by up to 51% in patients on high-intensity statin treatment [53,54]. The ongoing phase 3 Randomized Study to Evaluate the Effect of Obicetrapib on Top of Maximum Tolerated Lipid-Modifying Therapies (BROADWAY) trial will help ascertain the effect of raising HDL-c in the management of atherosclerotic disease.

Niacin is known to alter the lipid profile of patients with atherosclerotic disease by raising apo A1-containing HDL, reducing apoB lipoprotein particles, and inhibiting the synthesis of triglycerides in the hepatic cells, thus decreasing the secretion of LDL and VLDL particles [55]. Despite these favourable effects on lipoprotein particles, two large randomised trials did not demonstrate any reduction in cardiovascular events when patients were subjected to niacin compared to placebo [56,57]. Niacin was reported to increase the incidence of diabetes by 1.3%, as well as disturbing diabetic control by 3.7%. There was an unexpected increase in infection rate by 1.4% with the use of niacin [57]. Overall, this has led to limited use of niacin in the management of atherosclerotic disease.

## 4. Triglycerides Lowering Treatment

The relationship between hypertriglyceridemia and future adverse cardiovascular events is well established. Patients with mildly elevated triglycerides of more than 170 mg/dL had almost 50% increase in mortality [58]. A causal link between triglyceride-rich lipoprotein particles and the risk of ischaemic heart disease was suggested from previous Mendelian randomisation studies [59,60]. This was supported by statin trials suggesting a 10% reduction in the risk of death or recurrent myocardial infarction for each 1 mmol/L decrease in triglycerides level [61]. Treatments targeting hypertriglyceridemia have not been consistent in lowering future cardiovascular events.

Fibrates are one of the early therapies that lowered triglycerides via the nuclear receptor peroxisome proliferator-activated receptor α (PPARα). In statin-treated patients, fibrate did not reduce adverse cardiovascular events when compared to placebo [62,63,64]. Nonetheless, subgroup analyses have strongly linked a reduction in cardiovascular risk in response to fibrate treatment in patients with high triglycerides levels and low HDL-c, particularly if they have concomitant type 2 diabetes [65,66]. The Pemafibrate to Reduce Cardiovascular Outcomes by Reducing Triglycerides in Patients with Diabetes (PROMINENT) study assessed the use of pemafibrate, potent and selective PPARα modulator, in patients with type 2 diabetes, mild-to-moderate hypertriglyceridemia (200 to 499 mg/dL), and HDL-c ≤ 40 mg/dL [63]. The primary endpoint, defined as the composite of cardiovascular death, nonfatal myocardial infarction, ischemic stroke, and coronary revascularisation, was comparable to placebo [63].

Omega-3 fatty acids have historically been used to lower triglycerides. However, their data on reducing adverse clinical outcomes are conflicting [67,68]. The active metabolites of omega-3 fatty acids, docosahexaenoic acid (DHA) and eicosapentaenoic acid (EPA), have been assessed as adjunctive treatment to reduce future cardiovascular risk [69]. Whilst both fibrate and omega-3 fatty acids share a similar mechanism in reducing triglycerides by targeting PPARα, the former lacks the ability to increase the clearance of cholesterol remnants particles, resulting in no change and, sometimes, a slight increase in apo-B lipoprotein particles [69]. Early studies showed potential benefits in using omega-3 fatty acids. The JAPAN EPA Lipid Intervention Study (JELIS) assessed the role of high-dose purified EPA in 18,645 statin-treated patients with a total cholesterol ≥6.5 mmol/L [70]. There was almost 20% relative risk reduction in the composite endpoint of sudden cardiac death, fatal and non-fatal myocardial infarction, and other non-fatal events, including unstable angina pectoris, angioplasty, stenting, or coronary artery bypass grafting in patients receiving EPA [70]. The Reduction of Cardiovascular Events with Icosapent Ethyl–Intervention Trial (REDUCE-IT) highlighted almost 5% absolute risk reduction when subjecting statin-treated patients to an even higher dose of purified EPA (4 g/day) after a median follow-up of 4.9 years [67]. Notably, the clinical benefits were not related to the level of triglycerides but to the treatment of EPA, which may explain the inconsistency in the results of the studies assessing the role of omega-3 fatty acids [68,69]. There is a slight increase in the risk of bleeding and the incidence of atrial fibrillation associated with the use of high-dose EPA [67].

Emerging therapies targeting angiopoietin-like 3 protein (ANGPTL3) may add benefits to patients at increased cardiovascular risk. Blocking ANGPTL3 will disinhibit lipoprotein and endothelial lipase, leading to a reduction in triglycerides and LDL-c [16]. Evinacumab is a fully human monoclonal antibody inhibitor of ANGPTL3 and is currently approved for treating familial hypercholesterolemia [71]. It is administered intravenously every four weeks and has few reported adverse effects, including upper respiratory tract infections and flu-like syndromes. Like other intravenously administered drugs, evinacumab has the potential to cause severe allergic reactions or even anaphylaxis [72]. Vupanorsen is an antisense oligonucleotide that inhibits the liver synthesis of ANGPTL3. However, a recent clinical trial showed severe hepatic toxicity, questioning its safety clinical profile [71].

## 5. Lipoprotein-Specific Therapies

The fundamental steps in developing atherosclerosis are related to the accumulation and progression of cholesterol within the intima of vascular walls, triggering an inflammatory cellular cascade. This process is largely controlled by the influx of apo-B lipoproteins and the cholesterol efflux that is mediated by apo-A1. Cholesterol efflux capacity from macrophages is considered a metric of HDL functionality and is significantly determined by apo-A1 level [73]. Moreover, it has an inverse strong association with the development of atherosclerosis and the incidence of cardiovascular events [73,74]. Intuitively, increasing Apo-A1 resulted in an increase in ex vivo cholesterol efflux capacity in patients with acute myocardial infarction [75]. However, this did not translate into a reduction in cardiovascular events in the Rationale and Design of ApoA-I Event Reducing in Ischemic Syndromes II (AEGIS-II) study [76]. A novel approach has recently been proposed to decrease cholesterol by promoting its excretion. Loss-of-function variants of Asialoglycoprotein receptor 1 (ASGR1) were associated with low cholesterol and a reduction in cardiovascular disease [77]. ASGR1 deficiency was associated with increased cholesterol reverse transport via ABCA1 and ABCG5/G8 on HDL particles. The prevailing mechanism is related to stabilising LXRα, which subsequently upregulates ABCA1 and ABCG5/G8, enhancing cholesterol excretion to bile and faeces [77].

Since LDL-c is directly related to the development of atherosclerosis, reducing LDL may provide a therapeutic option to reduce future cardiovascular risks. Apolipoprotein B is one of the main components of LDL and VLDL particles and is central in packaging and redistributing cholesterol within the body. Antisense oligonucleotide treatments were developed to target hepatic synthesis of apoB100 and showed promising results by reducing apo-B up to 50% [78,79]. Mipomersen is a second-generation antisense that targets the coding region of the human apoB mRNA [79]. It is FDA-approved but has not yet been approved for use in Europe by the European Medicines Agency (EMA) [80]. A recent meta-analysis of conducted randomised clinical trials highlighted a mean LDL-c reduction of 26% in response to mipomersen [81]. However, mipomersen was associated with a few adverse effects, including elevation of liver enzymes, injection site reactions, and flu-like symptoms, which subsequently led to discontinuation in up to 20% of the patients investigated [81].

Numerous studies have demonstrated the importance of apoC-III in hypertriglyceridemia and its intrinsic proatherogenic effects [82,83]. ApoC-III is the current target for a new class of drugs aimed at lowering triglyceride levels and indirectly preventing cardiovascular incidents and pancreatitis episodes in patients with hypertriglyceridemia. The first of these drugs is volanesorsen, an antisense oligonucleotide that binds to the apoC-III mRNA and disrupts apoC-III translation, resulting in lower levels of chylomicrons and triglycerides [84]. Volanesorsen was associated with a 72% reduction in triglyceride levels, alongside pancreatic events, compared to placebo after three months [85]. The most common adverse effect of volanesorsen is injection site reaction; however, severe thrombocytopenia has also been observed [85]. Similar to volanesorsen, olezarsen is an antisense oligonucleotide targeting apoC-III, and through apoC-III translation disruption, chylomicrons and triglyceride levels are reduced. It is currently being studied, and initial results did not suggest thrombocytopenic risk [86].

Lipoprotein(a), Lp(a), is considered a variant of LDL as both contain apo-B lipoprotein, but Lp(a) carries lipoprotein (a), which is due to its structure is a promotor of inflammation and thrombosis. Lp(a) is genetically determined and not modified by exercise or diet. It is known to be an independent risk factor for atherosclerosis and cardiovascular events [87]. Therefore, it is currently being pursued as a therapeutic target to reduce future cardiovascular risk. Existing therapies such as statins are not associated with a clinically significant reduction in Lp(a) level. Likewise, PCSK9 inhibitors have a relatively marginal lowering effect, causing a 20–25% reduction in Lp(a) [31,32]. The HORIZON (Assessing the Impact of Lipoprotein(a) Lowering with pelacarsen (TQJ230) on Major Cardiovascular Events in Patients With CVD) outcomes trial is a phase 3 study that is currently assessing the role of pelacarsen, an antisense oligonucleotide, on reducing future cardiovascular risk [88]. Pelacarsen is a subcutaneously administered medication that binds to hepatocyte apo(a) mRNA, preventing the translation of apolipoprotein(a). It was associated with a significant reduction, up to 67%, in the level of circulating Lp(a) [89]. Olpasiran is another small-interfering RNA that reduces hepatic Lp(a) synthesis. In the phase 2 study, olpasiran was associated with a significant reduction in placebo-adjusted mean percent changes of up to 100% [90]. The outcomes of the phase 3 trial will be eagerly awaited.

## 6. Conclusions

**Figure 1:**
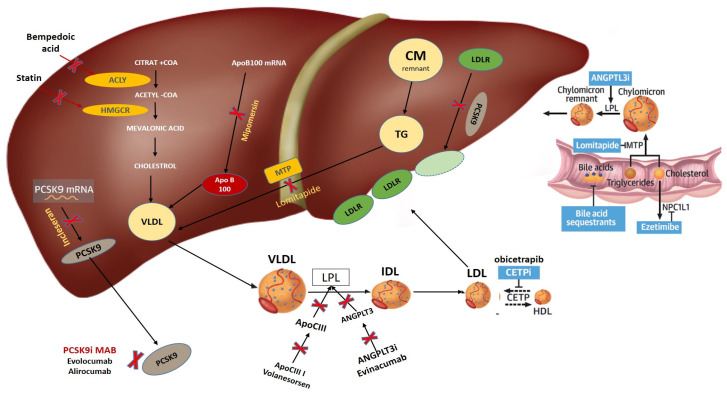
Mechanisms of non-statin drugs (ezetimibe, PCSK9 inhibitors, bempedoic acid, ApoB100 mRNA inhibitor, ApoCIII inhibitors, ANGPLT3 inhibitor, CETPi, MTP inhibitors, PCSK9 MAB, and PCSK9 mRNA inhibitor inclisiran). CoA, coenzyme A; ACLY, ATP citrate lyase; HMG-CR, HMG-CoA reductase; NPC1L1, Niemann-Pick C1-Like 1; mRNA, messenger RNA; LPL, lipoprotein lipase; MTP, Microsomal Triglyceride transfer protein; ANGPLT3i, Angiopoietin-Like Protein 3 (ANGPTL3) Inhibitors; PCSK9, proprotein convertase subtilisin/kexin type 9; PCSK9 MAB, proprotein convertase subtilisin/kexin type 9 monoclonal antibody; PCSK9 mRNA, proprotein convertase subtilisin/kexin type 9 messenger RNA; CETPi, cholesteryl ester transfer protein inhibitor; CM, Chylomicrons; VLDL, very low-density lipoprotein; IDL, intermediate-density lipoprotein; LDL, low-density lipoprotein; HDL, high-density lipoprotein; TG, Triglyceride; ApoCIIIi, Apolipoprotein C-III inhibitors; ApoB100 mRNA inhibitor, Apolipoprotein B100 messenger RNA inhibitors; ApoB100, Apolipoprotein B100; LDLR, low-density lipoprotein receptor.

Statins remain the cornerstone in the management of patients with atherosclerotic disease. However, a significant proportion of patients remain at increased risk, despite receiving maximal statin dose. This risk is related to sub-optimal LDL-c level but may also be derived from other lipoprotein-related risk. It is important to recognise the availability of other therapies that may reduce this residual lipid risk (Summary of landmark trials and mechanisms of available treatments is presented in Table 2 and Figure 1).

## Figures and Tables

**Table 1:** Pharmacological properties of most commonly used statins.

	Potency	Absorption	Bioavailability	Elimination Half-Life (h)	Metabolism
Atorvastatin	High intensity at a dose ≥40 mg	Lipophilic	12%	14	CYP 3A4
Rosuvastatin	High intensity at a dose ≥20 mg	Hydrophilic	20%	20	CYP2C9 and CYP2C19
Pravastatin	Low intensity	Hydrophilic	17%	1–2	OARPB3
Fluvastatin	Low intensity	Lipophilic	24%	1–2	CYP2C9
Simvastatin	Low intensity at dose ≤20 mg	Lipophilic	<5%	1–2	CYP 3A4

**Table 2:** Summary of major lipid-lowering treatment studies.

Study Name	Tested Treatment	Mechanism of Action of the Study Drug	Number of Patients	LDL-C Reduction	Primary Outcome
Improve IT	simvastatin–ezetimibe group vs. Simvastatin monotherapy	Reduces absorption of cholesterol from the small intestine	18,144	23–24%	absolute risk reduction, 2.0 percentage points; hazard ratio, 0.936; 95% confidence interval, 0.89 to 0.99; *p* = 0.016
SHARP TRIAL [91]	Simvastatin plus ezetimibe vs. Simvastatin and placebo	Reduces absorption of cholesterol from the small intestine	9270 with chronic kidney disease	-	17% reduction in major atherosclerotic events in the ezetimibe arm
EWTOPIA 75 [92]	Ezetimibe vs. usual care for patients above 75 years old	Reduces absorption of cholesterol from the small intestine	3796	25.9% vs. 18.5%	Ezetimibe reduced the incidence of the primary outcome by 34% (HR 0.66; *p* = 0.002). Additionally, composite cardiac events were reduced by 60% (HR 0.60; *p* = 0.039) and coronary revascularisation by 62% (HR 0.38; *p* = 0.007) in the ezetimibe group vs. the control group.
CLEAR Outcomes study [44]	Bempedoic acid group vs. placebo	Inhibition of adenosine triphosphate citrate lyase	13,970	26% vs. 10%	A primary endpoint event (death from cardiovascular causes, nonfatal myocardial infarction, nonfatal stroke, or coronary revascularization) occurred in (11.7%) of the bempedoic acid group and in (13.3%) of the placebo group (hazard ratio, 0.87; 95% CI, 0.79 to 0.96; *p* = 0.004)
FOURIER [31]	Evolucumab and statin vs. statin and placebo	Human monoclonal antibody (PCSK9), Inhibition of PCSK9 protein results in more LDL receptors available and increased uptake of LDL-C into cells	27,564	59% at the end of 48 weeks	The primary composite end point of cardiovascular death, myocardial infarction, stroke, hospitalization for unstable angina, or coronary revascularization. The primary endpoint occurred in (9.8%) in the evolocumab group and (11.3%) in the placebo group (hazard ratio, 0.85; 95% CI, 0.79 to 0.92; *p* < 0.001)
ODYSSEY OUTCOMES [32]	Alirocumab with high-intensity statin vs. high-intensity statin and placebo	Human monoclonal antibody (PCSK9), Inhibition of PCSK9 protein resulting in more LDL receptors available, and increased uptake of LDL-C into cells	18,924	54.7–62.7% more LDL reduction in the alirocumab group	A composite primary endpoint event occurred in (9.5%) in the alirocumab group and in (11.1%) in the placebo group (hazard ratio, 0.85; 95% confidence interval [CI], 0.78 to 0.93; *p* < 0.001).
ORION-3 [93]	Inclisiran	siRNA therapeutic inclisiran, which reduces hepatic production of (PCSK9), results in sustained reductions in LDL cholesterol	497	47.5%	LDL cholesterol was reduced by 47.5% (95% CI 50.7–44.3) at day 210 and sustained over 1440 days. The 4-year averaged mean reduction of LDL-C cholesterol was 44.2% (95% CI: 47.1–41.4),
Mendel-2 [94]	Both alirocumab and evolocumab have been studied as monotherapy vs. Ezetimibe.	Human monoclonal antibody (PCSK9), Inhibition of PCSK9 protein results in more LDL receptors available and increased uptake of LDL-C into cells	614	evolocumab group 57%, ezetimibe 18% compared to placebo.	Evolocumab yielded significant LDL-C reductions compared with placebo or Ezetimibe and was well tolerated in patients with hypercholesterolemia.
GLAGOV TRIAL [95]	Evolucumab and statin vs. statin and placebo to study the effect of Evolocumab on Progression of Coronary Disease in Statin-Treated Patients	Human monoclonal antibody (PCSK9), Inhibition of PCSK9 protein results in more LDL receptors available and increased uptake of LDL-C into cells	968	There was a marked decrease in LDL-C levels in the evolocumab group (Placebo 93 mg/dL vs. evolocumab 37 mg/dL; *p* < 0.001)	Percent atheroma volume (PAV) increased 0.05% with placebo and decreased 0.95% with evolocumab (*p* < 0.001), total atheroma volume (TAV) decreased 0.9 mm^3^ with placebo and 5.8 mm^3^ with evolocumab (*p* < 0.001).
The phase 3 Lp(a) HORIZON [88]	Pelacarsen	antisense oligonucleotides, small-interfering RNA-based therapies, gene editing, lowering of Lp(a)	8323	-	The estimated date of study end is May 2025
Pivotal trial [96]	Mipomersen	Antisense oligonucleotide targeting hepatic apoB100 mRNA	51 patients with Homozygote Familial Hypercholesterolemia	Mipomersen lowered LDL-C levels by 21% and apolipoprotein B levels by 24% compared to placebo	In addition, non-HDL-C was decreased by 21.6%, triglycerides by 17%, and Lp(a) by 23%, while HDL and apolipoprotein A-I were increased by 11.2% and 3.9% respectively.
REVEAL (Randomised Evaluation of the Effects of Anacetrapib Through Lipid-Modification) [97]	Anacetrapib	Cholesteryl ester transfer protein (CETP) inhibitor	30,449	HDL-C was increased by 104%, whereas LDL-C and apoB were reduced by 17% and 18%, respectively.	-
ANGPTL3 inhibitor phase 3 trial [98]	Evinacumab	fully human monoclonal antibody inhibits ANGPTL3	-	49%	-
REDUCE-IT [67]	Icosapent ethyl	highly purified and stable EPA ethyl ester, lower triglyceride levels and is used as an adjunct to diet	8179	-	The primary endpoint was a composite of cardiovascular death, nonfatal myocardial infarction, nonfatal stroke, coronary revascularization, or unstable angina, which occurred in 17.2% of the patients in the icosapent ethyl group, as compared with 22.0% of the patients in the placebo group (hazard ratio, 0.75; 95% confidence interval [CI], 0.68 to 0.83; *p* < 0.001)
Japan EPA Lipid Intervention Study (JELIS) [99]	eicosapentaenoic acid (EPA) of 1.8 gm daily plus statin vs. statin alone	Lower the triglyceride level	18,645	-	The risk of major coronary events was significantly lower, by 19%, in the group that received EPA plus statin therapy than in the group that received statin therapy alone.

## Data Availability

Not applicable.
